# Effective derivation of ventricular cardiomyocytes from hPSCs using ascorbic acid-containing maturation medium

**DOI:** 10.1080/19768354.2023.2189932

**Published:** 2023-03-24

**Authors:** Ji-eun Kim, Eun-Mi Kim, Hyang-Ae Lee, Ki-Suk Kim

**Affiliations:** a Dongguk University, Seoul, Republic of Korea; b Korea Institute of Toxicology, Daejeon, Republic of Korea

**Keywords:** Cardiomyocyte, ascorbic acid, albumin, Wnt signaling

## Abstract

Cardiomyocytes derived from human pluripotent stem cells (hPSCs) can be used in various applications including disease modeling, drug safety screening, and novel cell-based cardiac therapies. Here, we report an optimized selection and maturation method to induce maturation of cardiomyocytes into a specific subtype after differentiation driven by the regulation of Wnt signaling. The medium used to optimize selection and maturation was in a glucose starvation conditions, supplemented with either a nutrition complex or ascorbic acid. Following optimized selection and maturation, more cardiac Troponin T (cTnT)-positive cardiomyocytes were detected using albumin and ascorbic acid than B27. In addition, ascorbic acid enriched maturation of ventricular cardiomyocytes. We compared cardiomyocyte-specific gene expression patterns under different selection and maturation conditions by next-generation sequencing (NGS) analysis. Our optimized conditions will enable simple and efficient maturation and specification of the desired cardiomyocyte subtype, facilitating both biomedical research and clinical applications.

## Introduction

Human pluripotent stem cells (hPSCs) are a potentially an unlimited source of desired cell types, including cardiomyocytes (Takahashi et al. 2007; Burridge et al. 2012; Tohyama et al. 2013). hPSC-derived cardiomyocytes can be used in diverse applications, including disease modeling, cardiotoxicity screening, drug discovery, and basic research (Matsa et al. 2014). These applications require scalable, cost-effective, and reproducible production of cells under chemically defined conditions (Burridge et al. 2014) and feasible protocols for differentiating and maturing specific cardiac cell types (Liang et al. 2013; Landgren and Sartipy 2014). Over the past few years, several methods have been developed for generating cardiomyocytes from hPSCs, including embryoid body-based (Kattman et al. 2011) and monolayer-based differentiation protocols directed by addition of factors such as bone morphogenic protein 4 (BMP4), fibroblast growth factor 2 (FGF2), vascular endothelial growth factor (VEGF), and dickkopf-related protein 1 (DKK-1) (Laflamme et al. 2007; Paige et al. 2010). However, these methods have variable results and low efficiency in several cell lines and experimental replications (Paige et al. 2010). Regulation of the Wnt pathway by small molecules leads to nuclear accumulation of β-catenin, which is associated with Tcf/Lef (T-cell factor/lymphoid enhancer-binding factor) and activation of cardiac-related gene transcription, resulting in efficient differentiation of hPSCs into cardiomyocytes (Lian et al. 2012; Minami et al. 2012; Kim et al. 2022; Jung et al. 2022). Maturation into specific cell subtypes and purification of the resultant cells are critical steps for the successful use of hPSC-derived cardiomyocytes in applications such as high-throughput drug screening and toxicity testing.

Ascorbic acid is a small molecule that promotes induction of hPSC-derived cardiomyocytes (Takahashi et al. 2003; Fonoudi et al. 2015; Ivanyuk et al. 2015). Specifically, the addition of ascorbic acid during the maturation and purification of cardiomyocytes induces a unique subtype of cells that express cardiac-related genes, but not mesodermal markers (Cao et al. 2012). In other methods, differentiation by manipulation of the Wnt pathway in the presence of ascorbic acid yields a heterogeneous cell population that includes non-cardiac cell types such as fibroblasts, smooth muscle cells, and endothelial cells (Birket et al. 2013). Cardiomyocytes use glucose, fatty acids, and lactate as their main energy sources; in particular, the fetal heart uses lactate as its main energy source, whereas the adult heart uses fatty acids (Lopaschuk et al. 1992). Exposing differentiated cells to abundant lactate and glucose-depletion selectively purify cardiomyocytes (Tohyama et al. 2013).

In this study, we produced hPSC-derived cardiomyocytes by modulating the Wnt pathway with a small molecule inhibitor, and then purifying and maturing cardiomyocytes by taking advantage of their specific metabolic properties. We found that ascorbic acid promoted both structural and functional maturation of cardiomyocytes, as well as their differentiation from hPSCs. In addition, we analyzed gene expression patterns in the presence of ascorbic acid, either alone or in combination with other factors. This reproducible and efficient method, in which lactate-enriched conditions are used to selectively purify cardiomyocytes and ascorbic acid is used to promote maturation simultaneously, will facilitate clinical and pharmaceutical applications of hPSC-derived cardiomyocytes.

## Materials and methods

### Culture and differentiation of hPSCs into cardiomyocytes

hPSCs (H9) were maintained in E8 medium on vitronectin-coated plates. Cells were dissociated and plated using 0.5 mM EDTA in the presence of ROCK (Rho-associated kinase) inhibitor (Stemgent, U.S.A). To differentiate the cells into cardiomyocytes, the hPSCs were maintained on Matrigel-coated plates. Upon reaching almost complete conﬂuence (∼99%), the cells were treated for 24 h with 10 µM CHIR99021 (Selleckchem, U.S.A) in RPMI/B27-insulin (day 0 to day 1). To inhibit Wnt signaling, 5 μM IWP4 (Stemgent, U.S.A) was added on day 3 and removed during the medium change on day 5. Beating cells were observed starting on day 8–9, and the medium was replaced every other day.

### Selection and maturation of cardiomyocytes

To select and maturate cardiomyocytes, the differentiated cells were cultured for 10 days under one of two conditions: RPMI1640 without glucose (Life Technologies, U.S.A) supplemented with 4 mM sodium DL-lactate (Sigma-Aldrich, U.S.A) and B27-insulin (GIBCO, U.S.A); or RPMI1640 without glucose (Life Technologies, U.S.A) supplemented with 5 mM sodium DL-lactate (Sigma-Aldrich, U.S.A), 500 µg/mL human albumin (Sigma-Aldrich, U.S.A), and 211 µg/mL L-ascorbic acid (Sigma-Aldrich, U.S.A). The medium was replaced every other day.

### Immunostaining

Cells were ﬁxed with 4% (vol/vol) paraformaldehyde for 15 min, permeabilized with 0.5% (vol/vol) Triton X-100 in PBS for 5 min, blocked with 2% (wt/vol) BSA in PBS containing 0.1% (vol/vol) Triton X-100 for 20 min at room temperature, and then incubated overnight at 4°C with primary antibodies diluted in PBS with 0.1% (vol/vol) Triton X-100 and 2% (wt/vol) BSA. Immunoreactive cells were detected using Alexa Fluor 488–conjugated secondary antibody. Nuclear DNA was stained using Gold Antifade Reagent with DAPI (Invitrogen, U.S.A).

### Flow cytometry

Cultures were dissociated into single cells and then ﬁxed with 4% (vol/vol) paraformaldehyde for 20 min at room temperature, permeabilized with Perm buffer III (BD Biosciences, U.S.A) for 20 min at 4°C, and stained with primary and secondary antibodies in PBS plus 0.5% (vol/vol) FBS (Invitrogen, U.S.A) and 0.1% (wt/vol) sodium azide (Sigma-Aldrich, U.S.A). Data were collected on a FACSCalibur ﬂow cytometer (Becton Dickinson, U.S.A) and analyzed using FlowJo (FlowJo, LLC).

### Electron microscopy

Differentiated cells were plated on gelatin-coated glass coverslips. The clusters were ﬁxed overnight at 4°C in 2.5% (vol/vol) glutaraldehyde, and then postﬁxed with 1% (wt/vol) osmium tetroxide. Samples were dehydrated using an ethanol gradient and substituted with 100% (wt/vol) propylene oxide for 30 min, and then embedded with propylene oxide and Epon 812. Ultrathin 70 nm sections were stained with uranyl acetate and lead citrate. Samples were visualized on a Tecnai G2 Spirit scanning transmission electron microscope (FEI) at 120 kV. Images were acquired on a US4000 camera (Gatan, Inc.).

### Whole-cell patch-clamp recording for action potential analysis

Action potentials (APs) were recorded using the whole-cell patch-clamp technique with an Axopatch200B amplifier, an A/D converter, a Digidata 1440 digitizer, and pClamp10.2 software (Axon Instruments for Molecular Devices, USA). Patch pipettes were made from borosilicate glass capillaries (Clark Electromedical Instruments, UK) using a pipette puller (PP-830, Narishige, Japan). Resistance was 5–7 MΩ when filled with pipette solution. After 2 weeks of cardiac differentiation, hPSC-derived cardiomyocytes were transferred to four-well culture plates containing 0.1% gelatin-coated glass coverslips and maintained in a culture incubator at 37 °C for 2 weeks to allow further maturation.

For recording, a coverslip with adherent cells was placed into the recording chamber and perfused with an extracellular solution containing the following (in mM): 137 NaCl, 5.4 KCl, 1.8 CaCl_2_, 0.5 MgCl_2_, 10 HEPES, and 10 glucose (adjusted to pH 7.4 with NaOH). The internal solution contained (in mM) 120 K-Asp, 20 KCl, 5 NaCl, 2 CaCl_2_, 10 HEPES, 5 EGTA, and 5 Mg-ATP (pH 7.25). Typical APs in hPSC-derived cardiomyocytes were recorded in I = 0 mode. The spontaneous beating activity of single hPSC-derived cardiomyocytes was recorded, and only hPSC-derived cardiomyocytes that could beat stably were included in the analysis. Following stabilization of the AP waveforms, the average of five recorded APs was analyzed.

### RNA isolation

Total RNA was extracted from different samples using TRIzol (Invitrogen, U.S.A). The harvested samples were homogenized by several passages through a syringe needle in the presence of TRIzol solution. The resultant lysate was mixed with chloroform at a 1:5 ratio and centrifuged for 15 min at 12,000 *× g* at 4°C. The aqueous phase was transferred to a new tube and mixed with isopropanol at a 1:1 ratio. After centrifugation for 10 min at 12,000 *× g* at 4°C, total RNA was obtained after ethanol dehydration and dissolution by DEPC-D.W.

## Results

### Cardiomyocytes differentiated from hPSCs via temporal regulation of Wnt signaling

**Figure 1.**
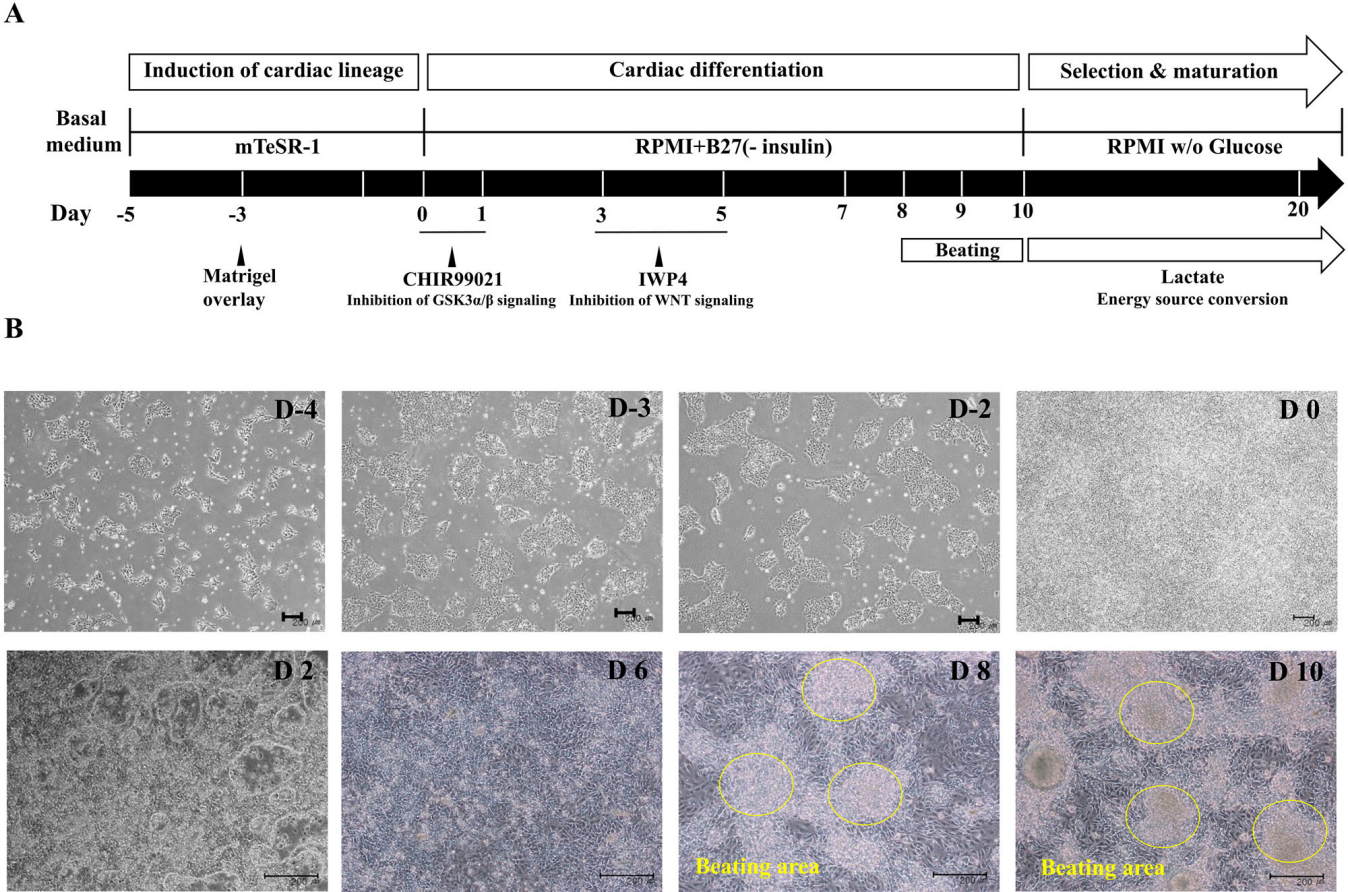
Cardiomyocyte differentiated from hPSC *via* temporal regulation of Wnt signaling. (A) Schematic representation of cardiac differentiation from hPSC *via* regulation of temporal Wnt signaling. (B) Cell morphology for differentiation process by date was assessed by phase contrast imaging. (Scale bar, 200 μm).

To induce cardiomyocyte differentiation, hPSCs were cultured on Matrigel-coated plates to confluence. Before exposure to differentiation protocol, the hPSCs consistently maintained pluripotency (Supplementary Figure 1). We attempted differentiation with a number of cell types to establish appropriate conditions (Supplementary Figure 2). To inhibit GSKα/β signaling, hPSCs were exposed to CHIR99021 for 24 h in RPMI medium with B27-insulin (Figure 1(A)) (Burridge et al. 2014; Fonoudi et al. 2015). Cells exposed to CHIR99021 exhibited a change in overall morphology (Figure 1(B)). Inhibition of GSKα/β signaling also induced the activation of the *Tcf/Lef* promoter, leading to genetically and consequentially mesoderm (Lian et al. 2012). After culture for an additional 2 days in RPMI medium with B27-insulin to produce precardiac mesoderm, we exposed the cells to IWP4, an inhibitor of Wnt signaling. After 2 days with IWP4, this generated cardiac progenitor cells (CPCs) (Sharma et al. 2015). The number of CPCs increased substantially after withdrawal of IWP4, and we observed beating cells after 8–9 days.

### Ascorbic acid with albumin promotes cardiomyocyte differentiation

**Figure 2.**
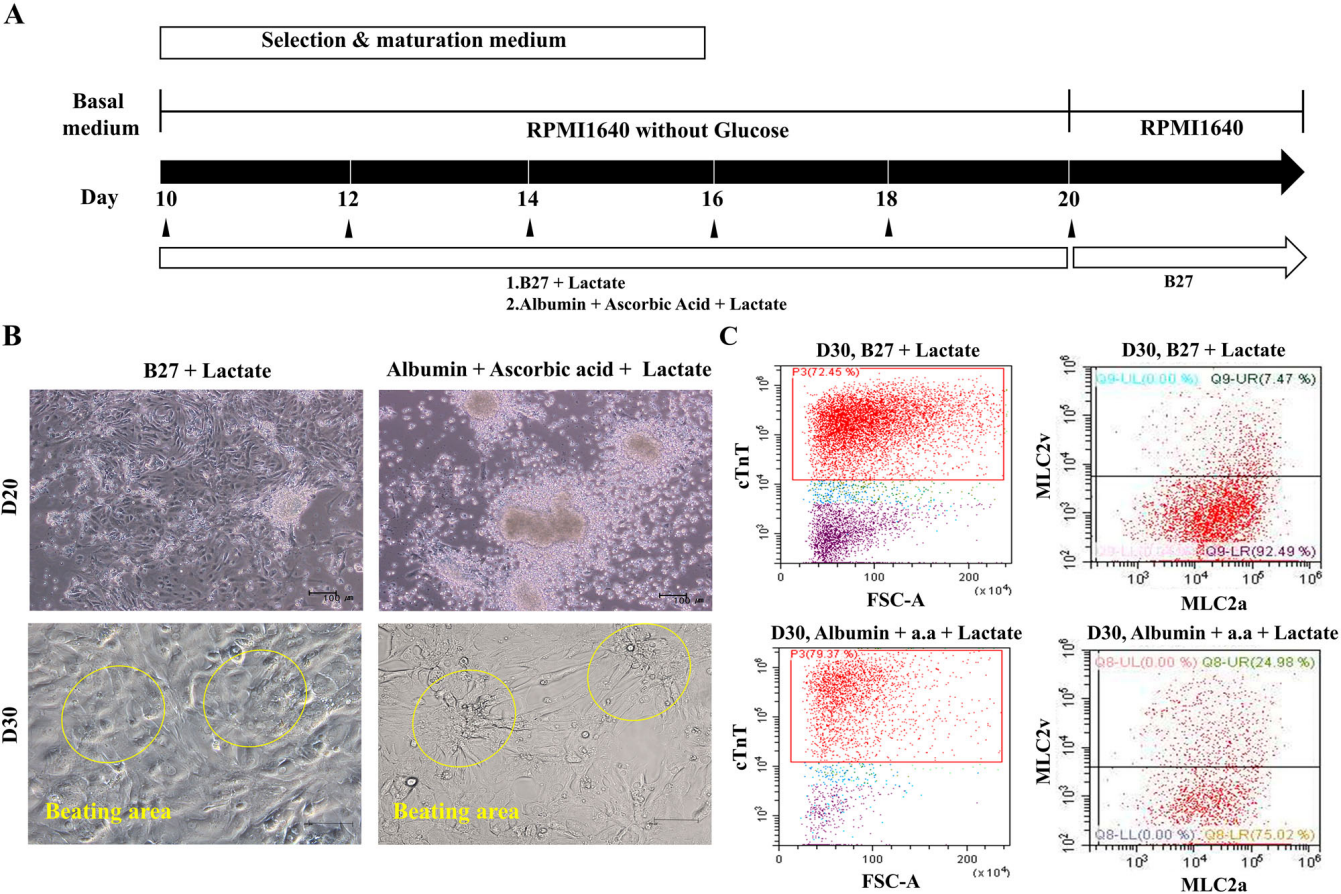
Ascorbic acid and albumin promote cardiovascular differentiation. (A) Schematic representation of cardiovascular differentiation via selection and maturation with or without ascorbic acid and albumin. (B) Phase contrast images show cell morphology at days 20 and 30 of selection and maturation medium. (Upper panel: Scale bar, 100 μm and lower panel: Scale bar, 200 µm) (C) The percentage of positive cells for MLC-2v, a marker for ventricular specification, in population of cTnT-positive cells maturated by medium containing albumin and ascorbic acid is higher than that in population of cTnT-positive cells by medium containing B27.

**Figure 3.**
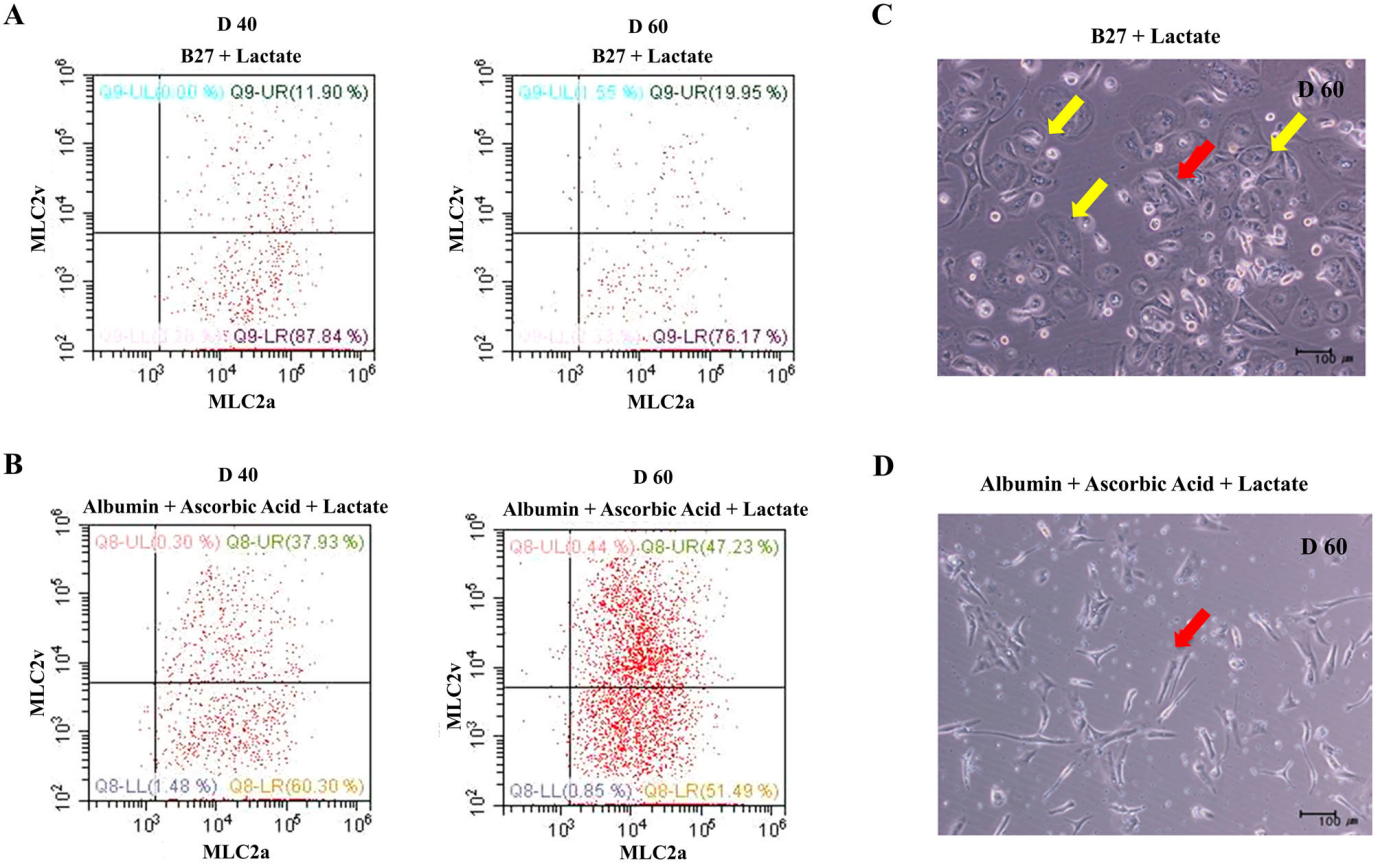
Quantitative analysis of hPSC-induced cardiomyocyte depending on the selection and maturation medium. (A and B) The differentiated cells were analyzed for MLC-2a and MLC-2v using flow cytometry at the indicated time points. The percentage of MLC2v-positive cells was higher in the presence of both ascorbic acid and albumin than B27 supplement alone. The percentage of MLC2v-positive cells was higher on day 60 than on day 40, whereas the proportion of MLC2a-positive cells decreased under both conditions. (C and D) The phase contrast images of the selected and matured cells depending on condition. Yellow arrow indicates the morphology of round shaped as atrial cardiomyocyte and red arrow indicates rod shaped as ventricular cardiomyocyte.

To optimize the selection and maturation of cardiomyocyte, differentiated cells (day 10 from onset of differentiation) were exposed to glucose-depleted, lactate-enriched RPMI medium with either B27 or albumin with ascorbic acid for 10 days, and then the selected and maturated cells were cultured in RPMI with glucose containing B27 (Figure 2(A)). We observed no difference in cell morphology between the two conditions on days 20 and 30 (Figure 2(B)). To compare the yields of cardiac differentiation, we analyzed the population of cTnT-positive cells by flow cytometry (Figure 2(C), left side). cTnT-positive cells constituted more than 70% of the total populations under both conditions, indicating that the identity of the supplement had no effect on the efficiency of cardiomyocyte selection and maturation (supplementary Figure 3). However, we did observe a difference in expression of the two major myosin light-chain 2 isoforms (MLC2a and MLC2v) in cTnT-positive cells on day 30 (Figure 2(C), right side), indicating diversity and maturity in the cardiomyocytes (Kubalak et al. 1994; Franco et al. 1999; Segev et al. 2005). In RPMI without glucose, with ascorbic acid and with albumin, more cTnT-positive cells expressed MLC2v, a marker of mature ventricular cardiomyocytes, than MLC2c, a marker of mature atrial cardiomyocytes (Figure 2(C), right side and lower panel). On days 40 and 60 after differentiation, we quantitatively assessed the differential expression of MLC2a and MLC2v (Figure 3). The percentage of MLC2v-positive cells was higher in the presence of both ascorbic acid and albumin than B27 supplement alone (Figure 3(A,B)). The percentage of MLC2v-positive cells was higher on day 60 than on day 40, whereas the proportion of MLC2a-positive cells decreased under both conditions (Figure 3(A,B), right side). Morphology was rod-shaped, corresponding to ventricular cardiomyocytes, or round, corresponding to atrial cardiomyocytes (Figure 3(C,D)). Thus, we confirmed that maturation of cardiomyocytes can be manipulated by addition of diverse supplements.

### Structural and functional characterization of hPSC-derived cardiomyocytes

**Figure 4.**
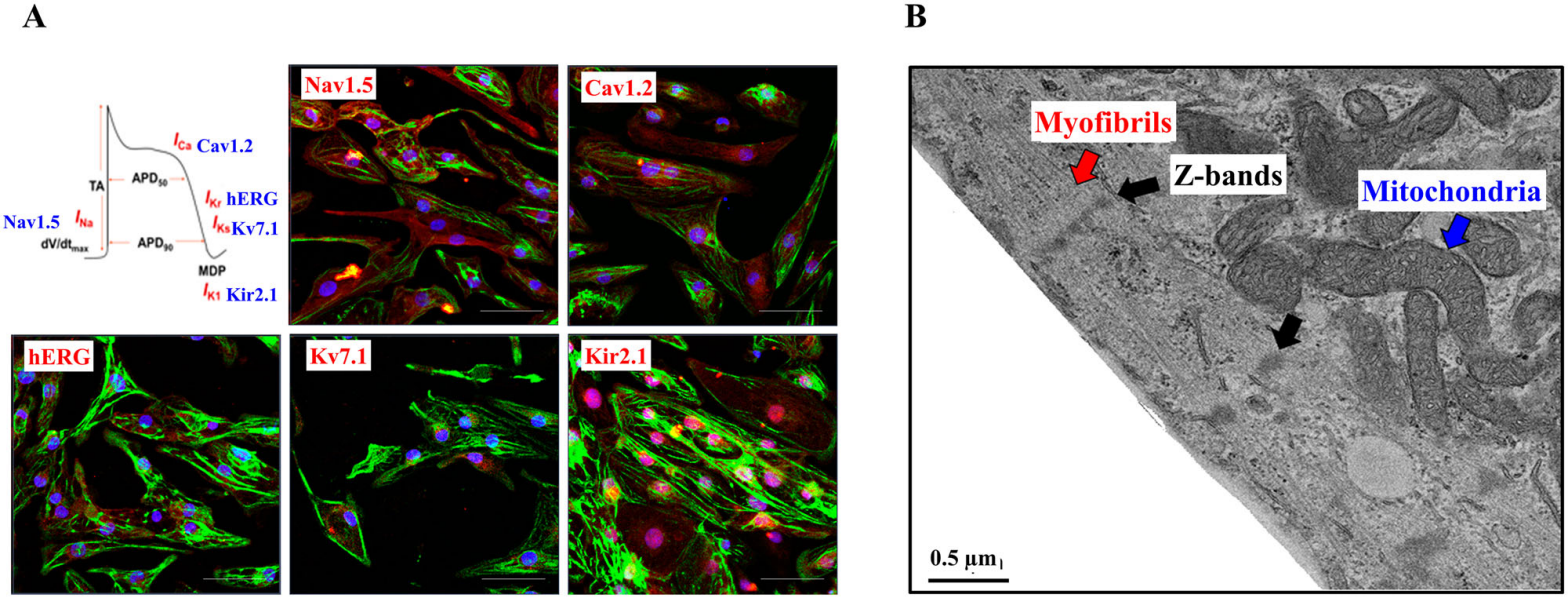
Structural and functional characterization of hPSC derived cardiomyocyte *via* Wnt signaling. (A) Immunostaining to various ion channels (Nav1.5, Cav 1.2, hERG, Kv7.1 and Kir2.1) reveals sarcomere organization. Nuclei and cTnT are shown in blue and green, respectively. (Scale bar, 200 μm) (B) An images of transmission electron microscopic shows myofibrils (red arrow) with Z-bands (black arrow) and mitochondria (blue arrow). (Scale bar, 0.5 μm).

To functionally characterize the cardiomyocytes selected and matured in glucose-depleted and lactate-enriched medium including ascorbic acid with albumin, we conducted immunostaining for subunits of ion channels, which are critical for cardiac function (Figure 4(A)). On day 30, this immunostaining revealed expression of Nav1.5 (a cardiac sodium channel) (Black and Waxman 2013), Cav1.2 (a cardiac calcium channel) (He et al. 2013), Kv7.1 (a delayed rectifier potassium channel), hERG (a cardiac potassium channel) (Aimond et al. 2005; Abbott et al. 2007; He et al. 2013), and Kir2.1 (a cardiac inward rectifying potassium channel) (Lange et al. 2003) in the cardiomyocytes. In addition, we assessed cardiac sarcomere organization by electron microscopy (Figure 4(B)). We observed myofibrillar bundles, transverse Z-bands, and enriched mitochondria, all of which are typical structural features of cardiomyocytes.

### Electrophysiological characterization of selected hPSC-derived cardiomyocytes

**Figure 5.**
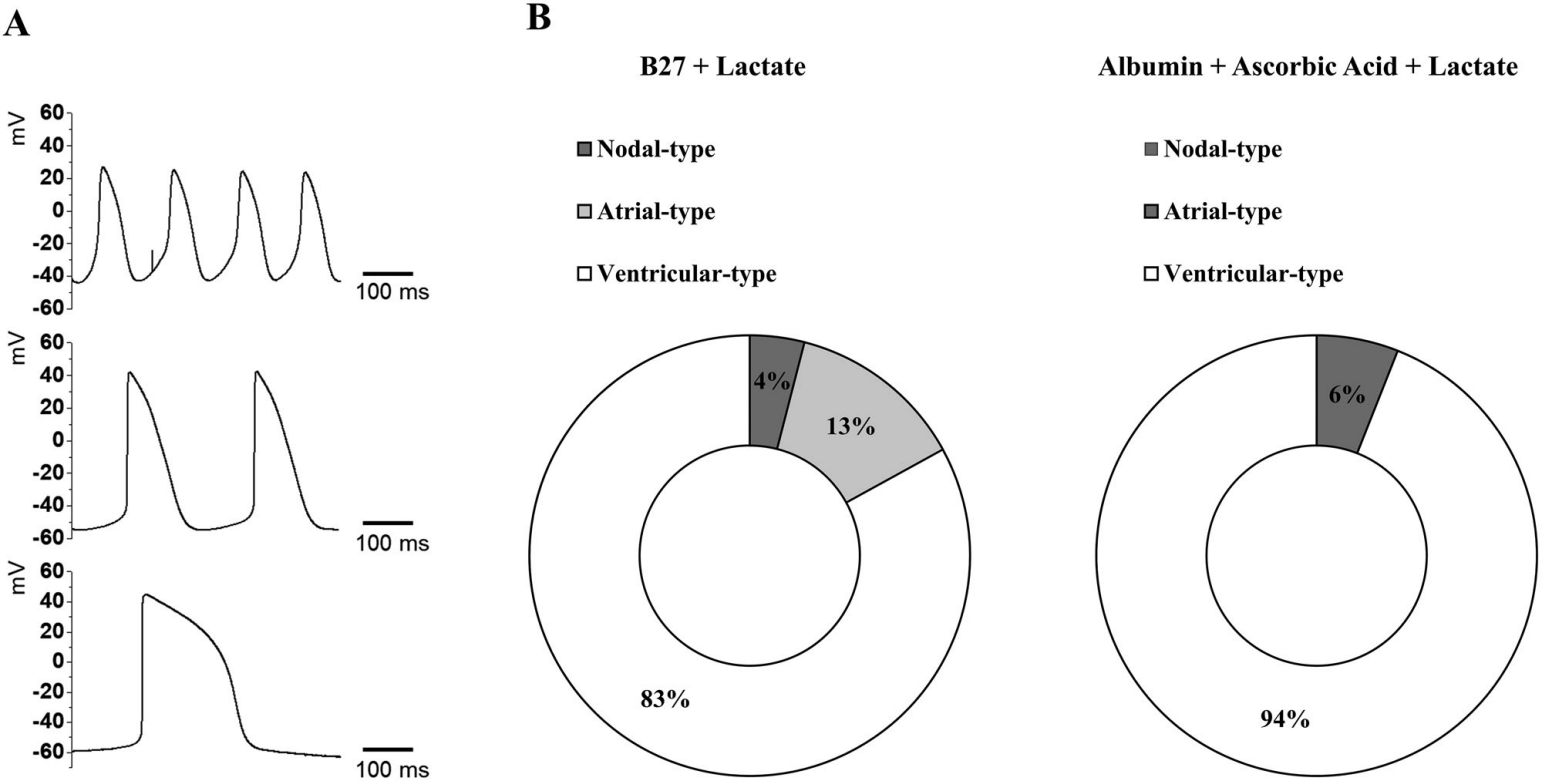
Action potentials (APs) of hPSC-derived cardiomyocytes (A) Representative images of three major types of AP traces observed in hPSC-derived cardiomyocytes. Nodal- (top), atrial- (middle), and ventricular-type (bottom) APs in hPSC-derived CMs. APs were recorded using intracellular sharp microelectrode recordings of single cells within monolayers. (B) Distribution of three different traces observed in B27 only (*n* = 24 cells) and ascorbic acid with albumin (*n* = 16 cells).

Using the patch-clamp technique, we measured spontaneous APs in contracting hPSC-derived cardiomyocytes. A schematic overview of APs and representative traces recorded in hPSC-derived cardiomyocytes are shown in Figure 5. Most cells exhibited ventricular-type APs in both the B27 and ascorbic acid with albumin groups; the percentage of ventricular-type APs was higher in the ascorbic acid with albumin group. Nodal-type APs were observed only in the B27 group, but the atrial-type APs were observed in both groups, with a higher percentage in the B27 group. Ventricular-type cells could be distinguished by their more negative maximum diastolic membrane potential (MDP) and rapid AP upstroke with long plateau phase. The absence of a prominent plateau phase is a characteristic of atrial-type APs, resulting in shorter AP duration (100 ms < AP duration at 90% repolarization (APD90) < 250 ms) than in ventricular-type APs (250 ms < APD90). Nodal-type APs had less negative MDP and slower AP upstroke. Table 1 compares the ventricular-type AP characteristics of hPSC-derived CMs in the B27 group and ascorbic acid with albumin group, alongside data for native human ventricular myocytes (hVMs) taken from the literature (Magyar et al. 2000).

**Table 1.** Action potential parameters in ventricular-type cardiomyocytes in native human, B27 + Lactate group and albumin + ascorbic acid + lactate group.

Cells	MDP (mV)	dV/dt_max_ (V/s)	APA (mV)	APD90 (ms)
Human VMs (Magyar et al. 2000)	−81.8 ± 3.3	215 ± 33	106.7 ± 1.4	351 ± 14
hPSC-derived VM^1^	−59.8 ± 0.8	14.9 ± 3.1	100.6 ± 1.1	306.6 ± 20.6
hPSC-derived VM^2^	−62.4 ± 2.4	94.2 ± 16.5	115.8 ± 1.8	302.5 ± 32.8

Note: The ventricular-type action potential parameters of native human in literature and both of B27 + lactate group and albumin + ascorbic acid + lactate group are summarized (mean ± SEM). VMs, ventricular myocytes;MDP, maximum diastolic membrane potential; maximum upstroke velocity (dV/dt_max_);action potential amplitude (APA);APD_90_, AP duration at 90_ % repolarization. ^1^B27 + lactate group, ^2^albumin + ascorbic acid + lactate group.

Next, we analyzed MDP, maximum upstroke velocity (dV/dt_max_), AP amplitude (APA), and APD90 in ventricular-type APs of H9-derived cardiomyocytes from the B27 group (*n* = 24 cells) and ascorbic acid with albumin group (*n* = 16 cells). The MDPs of ventricular-type CMs in the ascorbic acid with albumin group were still depolarized relative to the native hVMs, but still more negative than in the B27 group. dV/dt_max_ was lower in the ascorbic acid with albumin group (94.2 ± 16.5 V/s) than in the native hVMs (215 ± 33 V/s), but much higher than in the B27 group (14.9 ± 3.1 V/s). AP amplitudes were higher in the ascorbic acid + albumin group (115.8 ± 1.8 mV) than in hVMs (106.7 ± 1.4 mV) or the B27 group (100.6 ± 1.1 mV). APD90 was comparable to each other between the ascorbic acid with albumin group (302.5 ± 32.8 ms) and the B27 group (306.6 ± 20.6 ms), but slightly higher in native hVMs (351 ± 14 ms).

### Ascorbic acid promotes differentiation of ventricular cardiomyocytes

**Figure 6.**
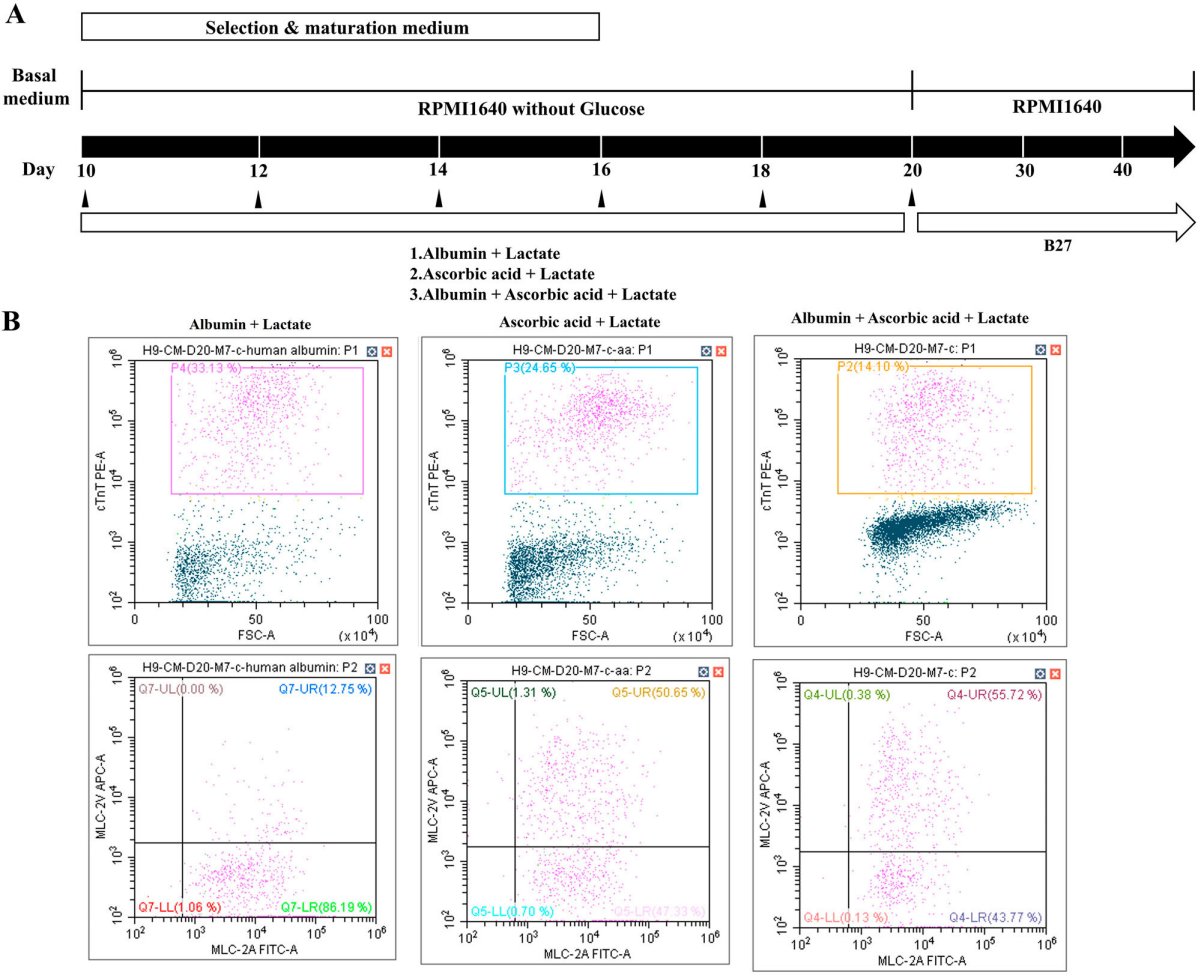
Ascorbic acid promotes differentiation of ventricular cardiomyocyte. (A) Schematic representation of cardiovascular differentiation via selection and maturation with or without ascorbic acid. (B) The percentage of positive cells for MLC-2v, a marker for cardiomyocyte and ventricular specification, is higher in the culture using a medium containing only ascorbic acid is higher than in other cultures of albumin or both. Lactate is added into all culture types.

We investigated whether ascorbic acid promotes differentiation of ventricular cardiomyocytes by examining three different formulations: albumin, ascorbic acid with albumin, and ascorbic acid in RPMI1640 without glucose (Figure 6(A)). The percentage of MLC2v-positive cells was highest in the ascorbic acid only group (Figure 6(B), middle panel), whereas the percentage of cTnT-positive cells was highest in the albumin condition (Figure 6(B), left panel). Thus, ascorbic acid promotes specification of ventricular cardiomyocytes to a greater extent than albumin alone (Figure 6(B), middle and right panels). In addition, ascorbic acid promotes specification of MLC2v-positive cells (i.e. ventricular cardiomyocytes) among cTnT-positive cells.

### Comparison of gene expression in cardiovascular differentiation via selection and maturation with or without ascorbic acid

**Figure 7.**
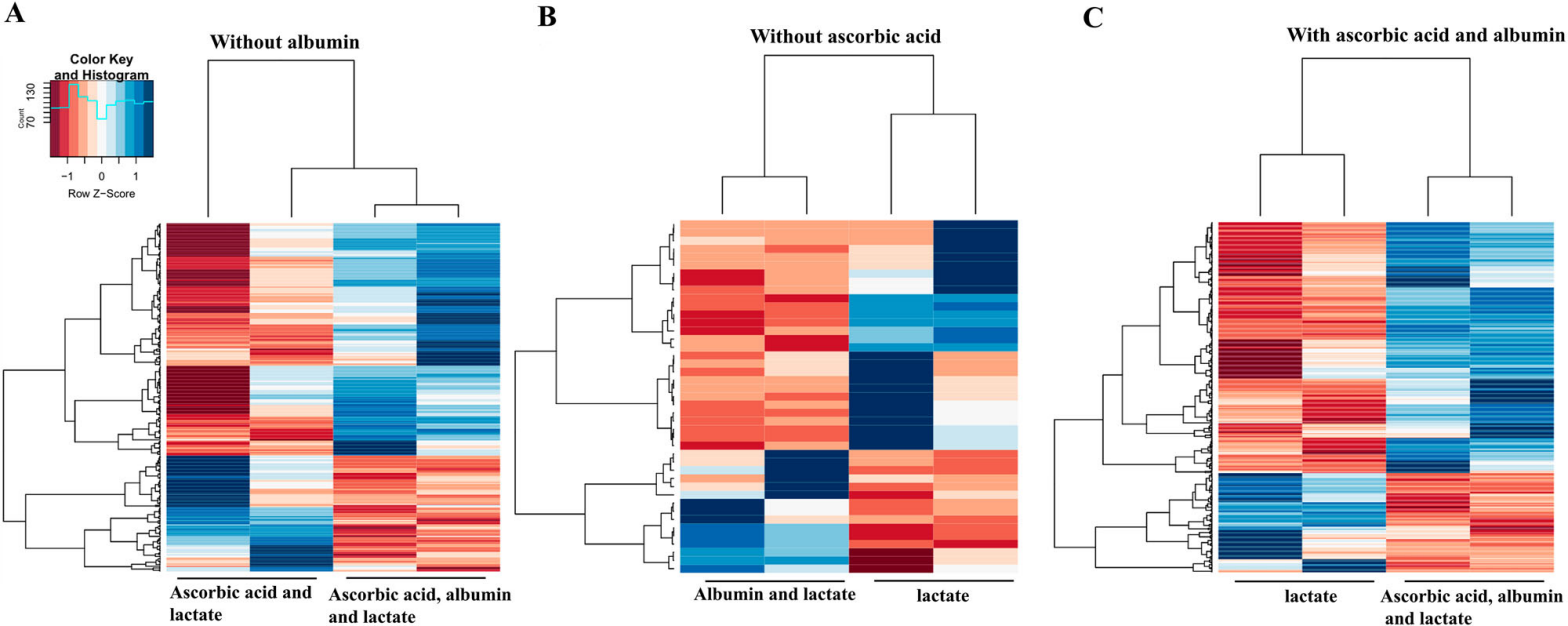
Heat map representing differentially expressed genes in conditions for selection and maturation of hPSC-derived cardiomyocyte. Blue indicates up-regulated expression, and red indicates down-regulated expression.

We then performed next-generation sequence (NGS) to compare gene expression among the albumin alone, ascorbic acid with albumin, and ascorbic acid alone conditions. We analyzed gene expressions of cardiomyocytes on 30 days after differentiation. Among the conditions compared, 34 genes were up-regulated in the albumin with ascorbic acid condition and 33 genes were down-regulated in the condition without ascorbic acid. Also, 10 genes were up-regulated and 18 genes were down-regulated in the only ascorbic acid condition (Figure 7(A–C)). This consequently yielded a list of up-regulated genes in selection and maturation medium containing albumin with ascorbic acid (Table 2).

**Table 2.** Genes up-regulated in hPSC-derived cardiomyocytes cultured in selection and maturation medium containing alubumin + ascorbic acid.

Genes	Full name
FHL2	Four and a half LIM domains 2
MYL2	myosin, light chain 2
DES	desmin
ANGPT1	angiopoietin 1
KIF1A	kinesin family member 1A
NDRG1	N-myc downstream regulated 1
SMIM1	small integral membrane protein 1
APOA4	Apolipoprotein 4
NMRK2	nicotinamide riboside kinase 2
COX6A2	cytochrome c oxidase subunit VIa polypeptide 2
RASL12	RAS-like, family 12
C14orf180	chromosome 14 open reading frame 180
COL14A1	collagen, type XIV, alpha 1
TBX3	T-box 3
IGFBP1	insulin-like growth factor binding protein-1

Note: The selected cardiomyocytes expressed genes related to cardiovascular development (ANGPT1, MYL2, COL14A, FHL2, SMIM1, IGFBP1 and TBX3) and muscle system processes (DES, KIF1A, RASL12, C14orf180 and NMRK2).

## Discussion

Cardiomyocytes derived from human pluripotent stem cells are useful *in vitro* cardiac models for toxicity screening and drug evaluations (Liang et al. 2013; Wang et al. 2014; Sharma et al. 2017). Accordingly, a reproducible, robust, and large-scale protocol for generating hPSC-derived cardiomyocytes is required for further development of pharmacology and toxicology platforms.

In this study, we demonstrated differentiation of hPSC-derived cardiomyocyte *via* regulation of canonical Wnt signaling without addition of growth factors. This protocol enabled hPSCs to differentiate into a population containing more than 70% cTnT-positive cells, indicating efficient and reproducible mesodermal induction through activation of Wnt/β-catenin signaling *via* Gsk3 inhibition (in this case, CHIR99021) (Lian et al. 2012; Burridge et al. 2014). Canonical Wnt signaling, a major regulator of hPSC-derived cardiomyocytes, triggers expression of developmental factors related to cardiomyocyte differentiation (Hoppler et al. 2014). After activation of Wnt signaling, inhibition of Wnt signaling with IWP4 induced highly efficient cardiomyocyte differentiation (Sharma et al. 2015). We achieved a cardiomyocyte yield of up to 70% when purification was conducted using lactate-enriched and glucose-starved conditions. Unlike selection by sorting, medium with lactate as the energy source allowed us to select cardiomyocyte in a simple and cost-efficient manner, taking advantage of the metabolic properties of these cells (Sharma et al. 2015). Selection and maturation of cardiomyocytes was achieved using B27 or albumin with ascorbic acid with conversion of the energy source. The percentage of cTnT-positive cells was higher in medium containing albumin with ascorbic acid than in medium including B27 alone. In addition, the percentage of ventricular cardiomyocytes among the cTnT-positive population was 3.3-fold higher in the albumin with ascorbic acid group than in the B27 group. Albumin helps cells to survive the process of selection and maturation (Francis 2010), and ascorbic acid promotes proliferation of CPCs (Takahashi et al. 2003; Cao et al. 2012) and large-scale generation of cardiomyocytes (Takahashi et al. 2003). Maturation into an adult-like phenotype and specific cardiac subtypes of hPSC-derived cardiomyocytes is necessary for toxicity evaluations using these cells (Yang et al. 2014). Ascorbic acid encourages the maturation of hPSC-derived cardiomyocytes by promoting accumulation of ECM components (Baharvand et al. 2005). In addition, the albumin with ascorbic acid condition enriched the ventricular subtype of cardiomyocytes during long-term culture. Our selected cardiomyocytes expressed cardiac ion channels and exhibited sarcomere organization, which are functional and structural features of mature cardiomyocytes, and 94% of their APs were ventricular-like. Under lactate-enriched conditions, specification of the ventricular subtype was stronger in medium containing only ascorbic acid. Ventricular cardiomyocytes, which are the major source of cardiac contractile forces, are important tools for *in vitro* assessment of drug toxicity (Cavero and Holzgrefe 2014; Chang et al. 2017). Accordingly, ascorbic acid promotes the specification of ventricular cardiomyocytes, as well as the proliferation of CPCs. Furthermore, selection and maturation medium containing ascorbic acid induced upregulation of genes related to cardiovascular development and muscle system processes.

## Conclusions

In this study, we generated highly mature and pure hPSC-derived cardiomyocytes by modulating the Wnt pathway with a small molecule inhibitor and exposing cells to glucose starvation. hPSC-derived cells generated with this technique expressed the specific markers of cardiomyocytes, as measured by FACS analysis, as well as other structural and functional properties of cardiomyocytes, including appropriate ion channel expression and typical cardiomyocyte organization. These hPSC-derived cardiomyocytes, selected and matured under conditions containing ascorbic acid, also exhibited normal electrophysiological properties, including different proportions of ventricular-type and atrial-type APs, but not nodal-type AP morphology. In an NGS analysis, we demonstrated that genes related to cardiovascular development and muscle system processes were upregulated in conditions containing ascorbic acid. This study not only demonstrates the role of ascorbic acid in maturation and purification of hPSC-derived cardiomyocytes, but also provides tools for drug evaluation related to cardiotoxicity.
